# Effect of the patient education - Learning and Coping strategies - in cardiac rehabilitation on return to work at one year: a randomised controlled trial show (LC-REHAB)

**DOI:** 10.1186/s12872-018-0832-2

**Published:** 2018-05-21

**Authors:** Birgitte Laier Bitsch, Claus Vinther Nielsen, Christina Malmose Stapelfeldt, Vibeke Lynggaard

**Affiliations:** 1 Department of Public Health, Centre for Rehabilitation Research, Aarhus University, P.P. Ørumsgade 11, 1B, 8000 Aarhus C, Denmark; 2 Department of Public Health, Section of Social Medicine and Rehabilitation, Aarhus University, Aarhus, Denmark; 3 DEFACTUM, Central Denmark Region, Aarhus, Denmark; 4 Cardiovascular Research Unit, Department of Cardiology, Regional Hospital West Jutland, Herning, Denmark

**Keywords:** Cardiovascular diseases, Return to work, Rehabilitation, Patient education, Learning, Coping

## Abstract

**Background:**

Personal resources are identified as important for the ability to return to work (RTW) for patients with ischaemic heart disease (IHD) or heart failure (HF) undergoing cardiac rehabilitation (CR). The patient education ‘Learning and Coping’ (LC) addresses personal resources through a pedagogical approach. This trial aimed to assess effect of adding LC strategies in CR compared to standard CR measured on RTW status at one-year follow-up after CR.

**Methods:**

In an open parallel randomised controlled trial, patients with IHD or HF were block-randomised in a 1:1 ratio to the LC arm (LC plus CR) or the control arm (CR alone) across three Danish hospital units. Eligible patients were aged 18 to ≤60 and had not left the labour market. The intervention was developed from an inductive pedagogical approach consisting of individual interviews and group based teaching by health professionals with experienced patients as co-educators. The control arm consisted of deductive teaching (standard CR). RTW status was derived from the Danish Register for Evaluation of Marginalisation (DREAM). Blinding was not possible. The effect was evaluated by logistic regression analysis and reported as crude and adjusted odds ratios (OR) with 95% confidence interval (CI).

**Results:**

The population for the present analysis was *N* = 244 (LC arm: *n* = 119 versus control arm: *n* = 125). No difference in RTW status was found at one year across arms (LC arm: 64.7% versus control arm: 68.8%, adjusted odds ratio OR: 0.76, 95% CI: 0.43-1.31).

**Conclusion:**

Addition of LC strategies in CR showed no improvement in RTW at one year follow-up.

**Trial registration:**

www.clinicaltrials.gov identifier NCT01668394. First Posted: August 20, 2012.

## Background

During the last decades, mortality from ischaemic heart disease (IHD) and heart failure (HF) has decreased due to improved primary and secondary prevention [1–3]. Thus, along with changed age composition, many people worldwide are living with these conditions which cause disability on several levels [4, 5]. Health promotion and risk factor reduction are typically managed in cardiac rehabilitation (CR) and CR is known to improve clinical outcomes [2]. Integrated patient education in CR programmes may also reduce fatal and/or non-fatal cardiovascular events and improve health related quality of life (HRQoL) [3, 6]. Patient education is recommended to focus on the individual’s personal resources rather than only increasing knowledge on disease management [7]. However, CR interventions have primarily been evaluated on clinical outcomes and less evaluated on the ability to promote level of function including return to work (RTW).

Work plays an important role for psychological and social wellbeing, and loss of productivity has economic costs for society [4, 8]. Clinical guidelines across nations therefore intend to cover vocational counselling in CR however, RTW internationally still seems to remain suboptimal [9, 10]. In Denmark it has been estimated that 21 and 25% of people with IHD and HF, respectively do not RTW 1 year after engaging in CR [11, 12]. Moreover, some patients struggle to balance workplace demands with the individual resources and health status and therefore experience recurrent sick leave episodes after RTW [13–15]. Problems in the work reintegration process has recently been emphasised since a study showed that detachment from employment was three-fold higher among post myocardial infarct patients 1 year after return to work compared with a matched population [16].

Personal resources as coping and self-care are important aspects in a successful RTW process [17]. CR-interventions aiming the ability to cope with and engage in everyday life evaluated on RTW have provided inconsistent results [18, 19]. Thus, the evidence of the pedagogical approaches and methods to promote the RTW process is unknown.

Learning and coping strategies (LC) is a patient education method that aims to facilitate personal resources through inductive teaching with a high level of patient involvement and includes supplement of individual clarifying interviews. The health professionals and experienced patients jointly perform the group based CR sessions [20]. The LC-REHAB trial was conducted in a hospital setting in Denmark, and aimed to assess the effect of LC strategies on various outcomes and have shown to promote patient adherence to CR [20, 21]. In the present trial it was hypothesised that the LC strategies also promoted RTW compared with usual CR by enabling patients to use acquired LC skills in the RTW process. Furthermore, that those receiving LC strategies would reduce the number of sick leave relapses by the gained insight of health condition and how to cope with that.

The primary aim was to assess the effect of adding LC strategies in CR on RTW 1 year after inclusion of patients diagnosed with IHD or HF. Secondary to assess if addition of LC strategies in CR reduced sick leave relapses during one-year follow-up.

## Methods

### Design

The trial was conducted on a subpopulation from the open randomised parallel group controlled trial, LC-REHAB [20]. Patients were randomly allocated to the intervention arm (LC strategies in addition to standard CR) or to the control arm (standard CR) in a 1:1 ratio stratified for hospital unit, gender and diagnosis (IHD or HF) in blocks of two to four using a web-based system [20]. The allocation procedure was generated independently by the research team. Additional eligibility criteria in the present trial were applied after randomisation to exclude patients assessed with permanent work disability at inclusion. Information on inclusion dates was retrieved from the LC-REHAB trial [20]. Follow-up was defined as the week in which the date equivalent to 12 months after inclusion appeared. The trial was conducted and reported according to the CONSORT standards – extension for randomised trials of non-pharmacological treatment [22].

### Patients and recruitment

**Fig. 1:**
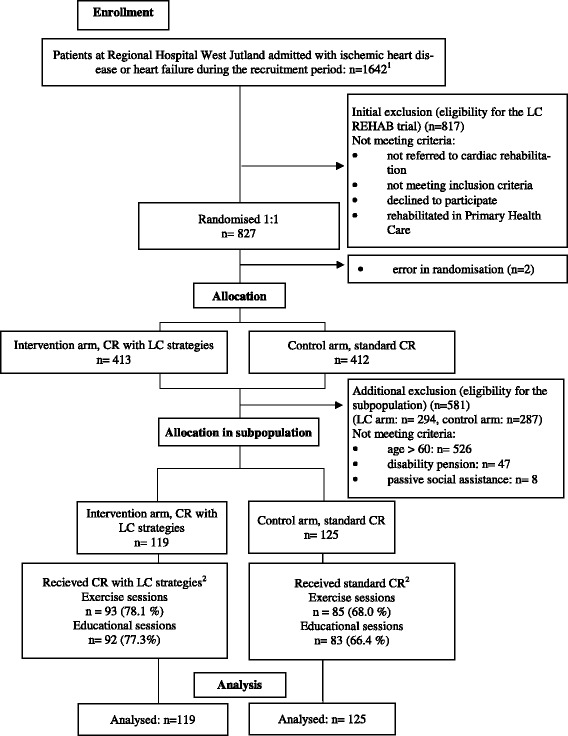
Flow diagram of patient selection for the LC-REHAB trial on RTW status. ^1^Patients (all ages, all employment status) assessed to require cardiac rehabilitation estimated from the Disease Management Program for Cardiovascular Diseases, Central Denmark Region [37]. ^2^Attending at least 75% of scheduled sessions corresponding to 18 exercise sessions and 6 educational sessions. The limit of 75% was set in accordance with recommendations for reducing mortality [38]

Patients were recruited between 30th November 2010 and 20th December 2012. Trial information was sent by postal mail to eligible patients referred to CR. Information of the trial was provided by telephone by the last author of this paper. Written informed consent, enrolment and randomisation were performed by health professionals at the CR units [20]. A total of 827 patients hospitalised for either IHD or HF were included in the LC-REHAB trial (Fig. 1). Two patients were excluded due to error in randomisation procedure. Of the remaining 825, 413 were randomised to the LC arm and 412 to the control arm.

Patients were enrolled at the CR unit and were eligible for the LC-REHAB trial if they were aged above 18, referred to, and motivated for CR after hospitalisation for IHD or HF. If the patients were diagnosed with both IHD and HF, they were classified as having HF. For specific ICD-10 codes included see the initial protocol for the LC-REHAB trial and previous study publication [20, 21]. The exclusion criteria were acute coronary syndrome within the last 5 days before inclusion; active peri-, myo-, or endocarditis; symptomatic and/or untreated heart valve disease; severe hypertension with blood pressure > 200/110 mmHg; other severe cardiac or extra cardiac disease; planned revascularisation; senile dementia, assessed as having poor compliance for participation in and completion of the trial; or previously participation in the trial.

Eligibility for the subpopulation was assessed after trial completion by pre-defined criteria independent of allocation arms. Inclusion criteria applied for the present trial were: aged > 18 to ≤60 years, being self-supported or received either State Educational grants, labour market-related benefits or health-related benefits that did not indicate a permanent job incapability except for patients in jobs on modified conditions (flexi jobs). Exclusion criteria were: aged above 60, receiving disability pension or passive social assistance that indicated pre-existing, long-term work disability. Patients were assessed eligible based on public transfer payments by the week of inclusion.

### Interventions

Patients in both arms received a phase II CR program lasting 8 weeks based on the national Danish guidelines on CR starting the first workday after inclusion [20, 23]. The programme was delivered in a hospital setting and consisted of group based sessions all lasting 1.5 h with a weekly education session and three exercise sessions per week. The content of the education sessions in both arms were split in eight topics, which were chosen in collaboration with experienced health professionals in CR and experienced patients who previously had undergone CR [20]. The topics were: Function and symptoms of the heart, lifestyle effects on the development of IHD and HF, emotional reactions, medication, tiredness, the importance of relatives or other networks, importance and types of exercise, and future life with a chronic disease. The education sessions were primarily managed by a nurse. Exercise sessions consisted of aerobe exercise and muscle strength training managed by a physical therapist.

Both arms received CR by the same pair of a nurse and a physiotherapist throughout the CR programme; the pairs were designated to either the intervention or control arm throughout the trial. Due to the nature of the intervention, blinding of health professionals or patients was not possible. Sessions in the two different arms were performed at different times of the day [20].

### Intervention arm (LC arm)

In addition to the described CR intervention, LC strategies took a situated, reflexive, and inductive approach to education and exercise. The rationale behind the pedagogical approach was based on theories behind LC strategies and was described in the initial protocol for the LC-REHAB [20]. The rationale was applied through practical implications consisting of: Two individual clarifying interviews (before and after CR), experienced patients as co-educators, material developed for each topic including background literature and questions to facilitate discussions. The approach was ensured by health professionals completing an 8 days competence-education in LC strategies with experienced patients participating the last 4 days, and 1 h evaluation meetings between the pair of health professionals and experienced patient once a week [20].

### Control arm (standard CR)

The CR programme in the control arm was the formerly used in the hospital units (standard CR) [23]. The rationale was not described and education and exercise consisted of structured deductive teaching. Identical pre-written, educational slide-shows were used as material for the education sessions.

### Outcomes

The primary outcome was RTW status at 1 year follow up. After trial completion, information on the RTW outcome was retrieved from DREAM, which is administered by The Danish Ministry of Employment [24]. The register includes all Danish citizens, who at some point since 1991 have received public benefits. Patients were identified in DREAM by their social security number. Each person is registered once a week with a code representing the type of transfer payment received that particular week [25]. For patients to be categorised as having a RTW status (yes) at 1 year follow up, the four consecutive weeks prior to the week of one-year follow up were either codeless (self-support) or had codes representing State Education Fund grants or flexi jobs. For the secondary outcome, each patient was categorised as having an event of RTW during one-year follow up (yes/no). The first event of four consecutive weeks of either self support or codes representing State Education Fund grants or flexi jobs was categorised as RTW (yes). The patients who experienced the event of RTW during follow up but were not registered RTW at 1 year follow up were identified. They were referred to as “relapsed patients”.

### Baseline characteristics

Baseline variables concerning: age, gender, height, weight, diagnosis (IHD or HF), presence of diabetes, smoking, civil status and former participation in CR were reported to dedicated databases in the LC-REHAB trial by the nurses at the CR units [20].

At the first CR session, the health professionals handed out self-reported questionnaires for assessing: presence of depression, level of education, and the annual household income [20]. Depression (yes/no) was assed by Major Depression Inventory (MDI) [26]. Level of education was classified by low, medium, or high. Household income was classified by low, medium, or high. For elaborated classification on level of education and household income, see the initial protocol [20].

Since work status prior to CR was assumed associated with RTW, additional information on employment was retrieved from DREAM after allocation to the trial arms [14]. No self support prior to CR was dichotomised (yes/no) encompassing whether the patient within 6 months prior to inclusion were self-supported for at least 1 week.

### Statistical methods

Descriptive statistics were used to compare the baseline characteristics – Chi-squared for the binary and categorical outcomes, and Studen’s t-test for the continuous. RTW status at 1 year was compared between the two arms using a logistic regression model. The result was presented both as unadjusted and adjusted odds ratios with 95% confidence interval (CI). Adjustments were carried out for the stratification variables: gender, cardiac diagnosis and hospital unit. Additional adjustment for age was performed, as age was expected to be associated with RTW [11]. To address the secondary aim of the trial, frequencies and percentages described the number of patients who experienced the event of RTW during follow up across trial arms. A comparison of the relapsed patients across arms was performed by chi-square test. All analyses were performed based on the intention-to-treat principle. Analyses were performed using Stata 14 software [27]. Data management was performed blinded from allocation.

### Power calculation

Suggesting a 14% difference in RTW proportions between arms estimated from Kruse et al. [11], the given sample size on 244 patients left this trial with a power on 89% testing on a 5% level of significance.

## Results

### Baseline data

A total of 526 patients were excluded due to age criteria, 55 due to receiving disability pension (*n* = 47) or passive social assistance (*n* = 8) on the date of randomisation. Two-hundred-and-forty-four patients were included for the present trial on RTW; 119 and 125 in the LC arm and control arm, respectively. Mean age was 51.8 years and the majority of the patients were men (77.0%). A small fraction (16.8%) had no self support prior to CR (Table 1). Baseline variables balanced across arms with no statistically significant differences between the two arms on baseline variables except for gender (males accounting for 83.2% in the LC arm vs. 71.2% in the control arm (*p* = 0.03)) (Table 1). Non-responders balanced across arms in baseline variables containing missing values (results not shown).

**Table 1:** Baseline characteristics of patients

	*n* = 244			
Variables	LC-arm (*n* = 119)		Control arm (*n* = 125)	
	*n* (%)		*n* (%)	
Age (year)				
20-29	0	(0.0)	2	(1.6)
30-39	6	(5.0)	5	(4.0)
40-49	32	(26.9)	37	(29.6)
50-60	81	(68.1)	81	(64.8)
Sex (male)	99	(83.2)	89	(71.2)^1^
BMI (kg/m^2^)				
BMI < 25	19	(16.0)	29	(23.3)
BMI 25-30	61	(51.3)	54	(43.2)
BMI > 30	39	(32.8)	42	(33.6)
Diagnosis				
IHD	97	(81.5)	104	(83.2)
HF	22	(18.5)	21	(16.8)
Diabetes				
No diabetes	98	(82.4)	113	(90.4)
Type I or Type II diabetes	21	(17.7)	12	(9.6)
Smoking				
Never	42	(35.3)	45	(36.0)
Former	53	(44.5)	48	(38.4)
Current	24	(20.2)	32	(25.6)
Lives alone (yes)	23	(19.3)	29	(23.2)
First time in CR (yes) (16% missing)^2^	84	(80.0)	83	(83.0)
Diagnosed depression (present) (16%missing)^3^	9	(8.7)	7	(6.9)
Education				
No or low	26	(21.9)	27	(21.6)
Medium	78	(65.6)	78	(62.4)
High	15	(12.6)	20	(16.0)
Household income (person/year) (23% missing)^4^				
0-249.000 DKK (low)	15	(16.0)	6	(5, 6)
250.000-699.999 DKK (medium)	55	(58.5)	60	(64.5)
≥ 700.000 DKK (high)	24	(25.5)	27	(29.0)
No self support prior to CR	19	(16.0)	22	(17.6)
Hospital unit				
Herning hospital	67	(56.3)	68	(54.4)
Holstebro hospital	48	(40.3)	51	(40.8)
Ringkøbing hospital	4	(3.4)	6	(4.8)

^1^*p*-value across arms was statistically significant (*p* = 0.03)

^2^Percent calculated accounting for missing values *n* = 39

^3^*n* = 38

^4^*n* = 57

### Outcomes

#### RTW status at one year

No patients died or emigrated during follow up. Regarding the primary outcome, a successful RTW status was observed for 64.7% (95% CI: 55.4- 73.2) in the LC arm, compared with 68.8% (95% CI: 59.9-76.8) in the control arm at 1 year follow up, corresponding to a crude OR 0.83 (95% CI: 0.49-1.42). Adjusting for gender, hospital unit, diagnosis, and age did not alter the result (OR = 0.76, 95% CI: (0.43-1.31), Table 2).

**Table 2:** RTW status at one-year follow-up with comparison of LC arm and control arm

	RTW status at one year^1^ n = 244					RTW during one year follow^4^ up n = 244		Relapsed patients at one year^5^	
	Yes n (%)	No n (%)	OR (95% CI)	OR adjusted (95% CI)^2^	OR adjusted (95% CI)^3^	Yes n (%)	No n (%)	Yes n (%)	*P*-value
Control arm	86 (69)	39 (31)	1	1	1	104 (83)	21 (17)	18	0.87
LC arm	77 (65)	42 (35)	0.83* (0.49-1.42)	0.78** (0.45-1.34)	0.76*** (0.43-1.31)	95 (80)	24 (20)	18	
Total	163	66				199	45	36	

^1^Frequencies and percentages analysed using logistic regression. Crude and adjusted odds ratios (OR)

^2^Adjusted for stratification variables: gender, diagnosis and hospital unit

^3^Adjusted for stratification variables: gender, diagnosis and hospital unit and age

^4^RTW during one year follow up by frequencies and percentages

^5^Comparison of relapsed patients across LC arm and control arm using chi-square test

* *P*-value = 0.50, **0.37, ***0.32

#### Comparison of RTW status at one year and RTW during follow up

Registering RTW during the 1 year follow-up resulted in a slightly increased proportion of patients that experienced the event of RTW: 80% (95% CI: 72-87) in the LC arm and 83% (95% CI: 76-89) in the control arm. Regarding the secondary outcome, Thirty-six patients were identified as relapsed patients during follow up and they were equally distributed across the LC arm and control arm (*p* = 0.87, Table 2).

## Discussion

The present trial showed that addition of LC strategies in hospital based phase II CR did not improve RTW status at one-year follow-up compared to standard CR. Nor did addition of LC strategies seem to reduce sick leave relapses during one-year follow-up.

Prior to this trial, comparable CR-interventions aiming at facilitating personal resources to improve RTW had consisted of patient-involving educational sessions, making an individual worksheet plan, including the role of the spouse, shared-decision making and a partnership-based approach [18, 19]. The evidence for these approaches and methods is inconsistent and rely on a sparse basis [18, 19]. In cancer rehabilitation no comparable educational interventions established effect regarding RTW neither [28]. Comparing the effect of patient educational approaches across studies is however complicated by methodical differences as well as heterogeneity in patients, compared interventions and CR-delivery.

The trial benefitted from being able to measure the effect of a patient education method developed from a specific pedagogical approach in patient education. Despite the lacking effect of the intervention on RTW, the intervention provides knowledge for further research in CR patient education. According to the first guidance for evaluating complex interventions by the Medical Research Council (MRC), lack of effect may reflect implementation failure rather than genuine ineffectiveness of the intervention and has been identified as problems concerning development, implementation, and evaluation [29].

The intervention in the LC arm was developed to aim at promoting the individuals’ personal resources rather than targeting multiple factors affecting level of function [30]. A more comprehensive approach has however been suggested with beneficial effects on RTW by both a review on CR and a Cochrane review on cancer rehabilitation [10, 28]. LC strategies in this trial lacked of involvement of contextual factors in general and workplaces in particular. The relapsed patients had jobs in health-care, service jobs and manual labour. Job type and support from the employer is found important for CR patients and recently contextual factors at the workplace and organisational practices have been identified to constrain the margin of manoeuvre in work reintegration [15, 31]. This may imply that the physical demanding jobs, that also are low level educational job types among relapsed patients are more difficult to reintegrate into after sick leave due to IHD or HF. Thus, the findings in this trial together with emerging evidence suggest development of interventions that foster accommodation and support from involvement of contextual factors like workplaces in integrated CR programmes [31].

The implementation of the theoretical understanding in LC strategies may be questioned, since it was not described to what extent the illness perspective on work resumption was approached by the health professionals in the CR sessions. In the present study low household income, low educational level, no self support prior to CR and higher age at baseline were all statistically significant risk factors for not adhering to the CR exercise sessions (results not shown). These socioeconomic factors are in line with frequently reported predictors for not only poor adherence to CR but also detachment from employment [16, 32]. It was likely to assume that the poorer adherence in high risk patients contributed to the absent effect of LC strategies. Implementation of future interventions to improve RTW should therefore ensure: adequate RTW-aimed interventions, include practical implications that in particular aim the process of RTW, and optimise adherence to CR for patients in high risk of detachment from employment.

Evaluation in this trial was done using an outcome that only accommodated a paid job or education and neglected possible enhanced participation in e.g. volunteer work or social relations. This standardised outcome may have conflicted with the individualised approach in LC strategies. Alternative evaluation of CR that measures more participation-related outcome might be relevant to reflect the aim of rehabilitation and furthermore to address the important well-known participation restrictions in patients with chronic IHD [5, 33].

### Study limitations and strengths

Information bias was considered minimal; DREAM has been validated against workplace-registered job attendance and long-term sick listing and found to have high sensitivity and specificity [34]. Classification of RTW based on transfer payments from DREAM has elsewhere been defined based on various numbers of weeks (ranging from 1 week to 5 weeks) [12, 13, 16]. The chosen definition of four consecutive weeks in this trial might have affected the frequencies of RTW but was not expected to be differentiated between arms. Selection bias was furthermore not considered as there was complete follow up; therefore, threats to the internal validity of the trial were not assumed.

The trial was carried out in western Denmark where the population in general is lower educated than the total population of Denmark and the trial enrolled patients with HF [35]. Both level of education and living with HF are associated with increased risk of not returning to work and may have caused the overall lower RTW proportion (61-73%) in this trial compared to other studies [11, 12, 16].

According to the power calculation, a 14% difference in RTW was expected between the two trial arms; however the trial detected a 4 percentage point difference. It was expected that lacking practical implications in the LC strategies for improving RTW rather than a low sample size was the reason of no difference.

The estimate may have been affected towards the null hypothesis as mutual interaction between the arms was plausible due to lack of blinding of the health professionals. Moreover, patient education was delivered in both arms and the effect of the patient education in the control arm may have contributed to even out the effect of LC strategies.

Approximately, 50% of the population with IHD and HF asked to participate declined to participate in the trial [21]. However, no knowledge of the patients that declined was accessible and it was thus unknown if selection of the patients was present at enrolment. This caused limitation of the generalisability of the results and the trial was not able to provide answers about the effect on the total population of people with IHD or HF.

Temporal and contextual factors affect the ability to RTW and influence the external validity of the outcome measure. Also, this means that comparing results in an international context should be done carefully due to heterogeneity in RTW definitions and occupational systems.

## Conclusion

Addition of LC strategies in CR showed no improvement of RTW compared to CR alone after 1 year. Implications for further development and research of patient education methods in CR to improve RTW are: involvement of contextual factors in development of the intervention, and implementation that ensures practical implications targeting RTW like workplace involvement and job type. Lastly, evaluation should address the interventions’ ability to improve participation among patients living with IHD and HF.

## Abbreviations

CR: Cardiac rehabilitation
DREAM: Public transfer payments register
HF: Heart failure
IHD: Ischaemic heart disease
LC: Learning and coping
RTW: Return to work
